# Aerobic Conditions Enhance the Photocatalytic Stability of CdS/CdO_*x*_ Quantum Dots

**DOI:** 10.1002/chem.201802353

**Published:** 2018-06-27

**Authors:** David W. Wakerley, Khoa H. Ly, Nikolay Kornienko, Katherine L. Orchard, Moritz F. Kuehnel, Erwin Reisner

**Affiliations:** ^1^ Christian Doppler Laboratory for Sustainable SynGas Chemistry Department of Chemistry University of Cambridge Lensfield Road Cambridge CB2 1EW UK

**Keywords:** hydrogen, oxygen inhibition, oxygen tolerance, photocatalysis, quantum dots

## Abstract

Photocatalytic H_2_ production through water splitting represents an attractive route to generate a renewable fuel. These systems are typically limited to anaerobic conditions due to the inhibiting effects of O_2_. Here, we report that sacrificial H_2_ evolution with CdS quantum dots does not necessarily suffer from O_2_ inhibition and can even be stabilised under aerobic conditions. The introduction of O_2_ prevents a key inactivation pathway of CdS (over‐accumulation of metallic Cd and particle agglomeration) and thereby affords particles with higher stability. These findings represent a possibility to exploit the O_2_ reduction reaction to inhibit deactivation, rather than catalysis, offering a strategy to stabilise photocatalysts that suffer from similar degradation reactions.

Clean‐burning, renewable H_2_ fuel can in principle be generated effectively through solar‐driven proton reduction coupled to water oxidation as an abundant source of electrons.^1^ Alternatively, this reaction can be undertaken through the oxidation of organic species, either in the form of biomass‐derived substrates, such as EtOH, MeOH, glucose or lignocellulose,^2^, ^3^, ^4^ or through selective organic oxidation reactions to generate higher‐value products.^5^ Semiconductor particles are particularly well‐suited to perform the underlying reactions behind artificial photosynthesis and as such, rapid light‐driven H_2_ evolution has been reported for numerous metal oxide, sulfide, selenide and nitride‐based semiconductors.^6^, ^7^

Due to the ubiquity of O_2_ in the atmosphere, as well as its production in the water‐splitting reaction, a proton‐reduction catalyst must be able to tolerate its presence during activity.^8^, ^9^, ^10^ To date, little research has considered the effect of O_2_ on semiconductor‐driven H_2_ evolution and only few reports are available on O_2_‐tolerant molecular proton‐reduction catalysis.^11^, ^12^, ^13^, ^14^ Several strategies have therefore sought to protect proton‐reduction photocatalysts from O_2_ to allow catalysis to proceed. For example, deposition of thin layers of metal oxides, such as Cr_2_O_3_ and SiO_*x*_/TiO_*x*_,^15^, ^16^ on the surface of a proton reduction catalyst can selectively prevent diffusion of O_2_ to the catalyst, albeit under low levels of O_2_ (<1 atm of pressure).

Previously reported systems have shown that proton reduction catalysts fall into two groups: O_2_ sensitive, where a catalyst is irreversibly damaged by O_2_, or O_2_ tolerant, where a catalyst is able to function under O_2_, but at a reduced rate (Scheme 1).^8^ Nevertheless, the intrinsic oxidising nature of O_2_ does not need to be considered exclusively as a disadvantage and methods that use O_2_ to stabilise activity can be envisioned. O_2_ reduction is thermodynamically more facile than proton reduction and its presence in solution therefore offers a route to prevent a photocatalyst from a reductive deactivation pathway. Cu^I^Rh^III^O_2_ and Cu^I^Fe^III^O_2_ delafossite‐structured H_2_‐evolving photocathodes were previously demonstrated to operate most effectively under air using this strategy.^17^, ^18^ In these examples, Cu^0^ accumulates under inert conditions, which can be avoided through the introduction of O_2_, thereby increasing the electrode stability.

**Scheme 1:**
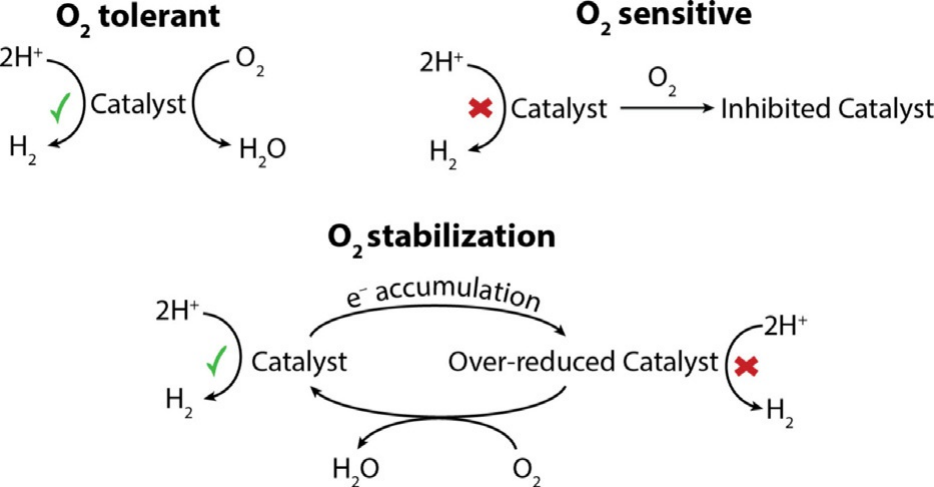
The potential influence of O_2_ in catalytic proton reduction.^8^

In this study, we demonstrate that O_2_ can be used to stabilise activity in colloidal “one‐pot” photocatalytic schemes and that even an improvement in catalytic H_2_ evolution performance can be achieved with CdS quantum dots (QDs). CdS QDs are nanocrystals below 10 nm in diameter that have previously demonstrated excellent photophysical properties for light‐driven proton reduction in the presence of sacrificial electron donors, catalysing this reaction at benchmark rates.^19^

The photocatalytic H_2_ evolution activity of CdS has been reported to drop by 20 % under 21 % O_2_ when compared to anaerobic conditions.^20^ This observation can be assigned to the competitive reduction of O_2_ versus protons, as seen for other O_2_‐tolerant catalysts (Scheme 1); however, we show that by encouraging sufficiently fast H_2_ evolution, over‐accumulation of reduced Cd^0^ at the particle occurs. Addition of O_2_ to this system precludes Cd^0^ formation and affords rapid and stable light‐driven H_2_ evolution.

Capping‐ligand‐free CdS QDs were synthesised with a diameter of 4–5 nm,^21^ as confirmed by transmission electron microscopy (TEM, Figure 1 a, see Supporting Information for experimental details).^3^ Photocatalytic experiments were undertaken by combining the QDs with Co(BF_4_)_2_ (0.25 mm), as a co‐catalyst for the proton‐reduction reaction, and MeOH (10 m), as a sacrificial electron donor, in a sealed photoreactor that was irradiated with simulated solar light (AM 1.5 G, 100 mW cm^−2^) at 25 °C. The production of H_2_ was analysed at designated time intervals by headspace gas chromatography (Table S1 in the Supporting Information). Control experiments showed that no H_2_ was produced in the dark or in the absence of CdS (Table S2).

**Figure 1:**
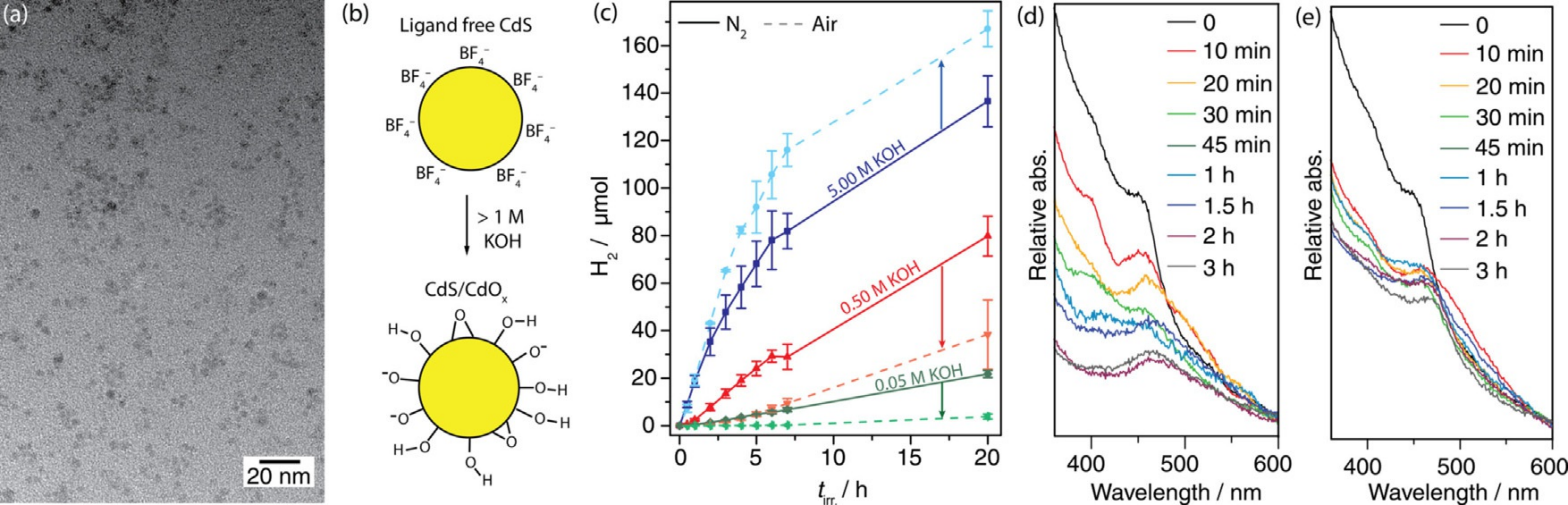
(a) TEM image of ligand‐free CdS QDs. (b) Illustration of CdS/CdO_*x*_ formation from particles of ligand‐free CdS‐BF_4_. (c) Photocatalytic H_2_ production (AM 1.5 G, 100 mW cm^−2^) at 25 °C from CdS QDs (0.5 μm) in various concentrations of aqueous KOH (2 mL) containing MeOH (10 m) in anaerobic (solid traces) or aerobic (dashed traces) conditions with 0.25 mm Co(BF_4_)_2_. (d) UV/Vis spectra of CdS/CdO_*x*_ QDs at designated intervals after photocatalysis in aqueous KOH (2 mL, 5 m) containing MeOH (10 m) in the presence of Co(BF_4_)_2_ (0.25 mm) under anaerobic conditions (N_2_). (e) The aerobic equivalent of the experiment in (d).

In highly‐alkaline solutions (>1 m KOH), a surface layer comprised of CdO/Cd(OH)_2_ (CdO_x_, Figure 1 b), forms on the particles, creating CdS/CdO_*x*_ QDs.^3^ This layer is believed to enhance the rate of photocatalysis. As such, in anaerobic conditions (Figure 1 c, solid lines), CdS QDs displayed the highest rate of H_2_ evolution in 5 m KOH, as previously reported.^22^ However, the activity is not stable and slows down after only a few hours (Figure 1 c). This coincides with the formation of a black precipitate in the solution and a loss of the CdS absorption peak in the UV/Vis spectrum (Figure 1 d). In contrast, in 0.5 m and 0.05 m KOH the rate of H_2_ evolution was slower, but did not drop significantly over time and the solution remained yellow. Electron transfer from photoexcited CdX semiconductors (X=S, Se) has previously been proposed to originate from surface Cd^0^ sites,^23^ and the black colour was therefore assigned to over‐formation of Cd^0^ (see below for characterisation).

Photocatalysis was subsequently performed in a closed vessel with an aerobic headspace to determine whether the extent of Cd^0^ formation could be reduced using O_2_. Depending on the rate of H_2_ evolution, the presence of O_2_ had differing effects (Figure 1 c, dashed lines). In 0.5 m and 0.05 m KOH, the introduction of O_2_ led to a decrease in photocatalytic performance by a factor of 48 and 82 %, respectively. This effect is expected, as the O_2_ reduction reaction competes with the desired proton reduction reaction (Scheme 1). In 5 m KOH, the aerobic activity was surprisingly enhanced relative to anaerobic conditions, with a reduced formation of the black precipitate. Figure 1 e illustrates the change in the UV/Vis spectrum of an aerobic sample over time, showing the retention of the CdS absorption band over 3 h of photocatalysis. In this sample, an eventual slowdown of the catalysis was visible after 6 h (Figure 1 c), which was a result of consumption of O_2_ within the vessel headspace.

Transient absorption (TA) and Raman spectroscopy were subsequently employed to gain further understanding of the processes. The relationship between the pH and electron/hole dynamics was first probed under aerobic conditions by TA. Figure 2 a shows the TA spectrum of CdS/CdO_*x*_ QDs in 10 m, 0.1 m and 0 m KOH with EtOH as an electron donor at a 1.5 ps delay, normalised to unity. The band‐edge bleach of the CdS/CdO_*x*_ QDs appears at ≈488 nm, arising from electrons being excited to the conduction band. The spectra also display a second negative peak at 600–700 nm, which is tentatively assigned to surface‐state traps.^22^

**Figure 2:**
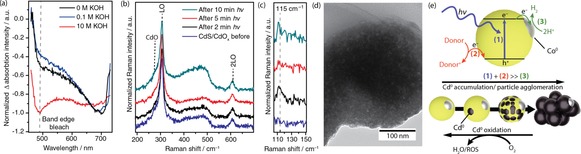
(a) UV/Vis transient absorption spectra of CdS/CdO_*x*_ in varying concentrations of KOH with 1 m EtOH at a 1.5 ps delay, showing the band‐edge bleach at 490 nm normalised to unity. (b) Raman spectra of CdS under Ar after irradiation with 1 mW of a 413 nm laser line for various time intervals. The spectra show the CdS LO and 2LO region of CdS/CdO_*x*_ (10 μm) in 10 m KOH (1 mL) with EtOH (1 mL) recorded using a 514 nm laser line (5 mW) with a 30 s accumulation time. (c) Raman spectra from (b) at lower wavenumbers, showing the emergence of a peak assigned to Cd^0^ formation at 115 cm^−1^. (d) TEM image of CdS/CdO_*x*_ QDs after 50 min of photocatalysis in 10 m KOH (1 mL) and MeOH (1 mL) in the presence of Co(BF_4_)_2_ (0.25 mm). (e) Illustration of the photocatalytic processes behind H_2_ evolution on CdS/CdO_*x*_ QDs and their relation to particle agglomeration and O_2_‐driven stabilisation.

Given the lower magnitude of the CdS signal in 0 m and 0.1 m KOH relative to CdS in 10 m KOH (see Figure S4 for absolute absorbance data), we propose that fewer photogenerated electrons are available due to ultrafast trapping and recombination pathways in the less alkaline conditions. In addition, a proportionally stronger bleach signal at 675 nm indicates that a larger fraction of electrons is trapped in the surface states rather than the conduction band at lower pH. The growth of a CdO_*x*_ layer on the CdS surface may therefore increase the efficiency of photocatalysis by ensuring that electrons remain in the conduction band, rather than other trap states. The greater accumulation of electrons in the conduction band at high pH is likely to lead to a greater propensity for proton reduction, as well as the self‐reduction of CdS to Cd^0^.

Raman spectroscopy under anaerobic conditions supports the proposed formation of Cd^0^ after electron accumulation. QD solutions in 10 m KOH with EtOH were irradiated with a 413 nm laser (1 mW) over various time intervals and spectra were recorded using a 514 nm excitation beam (Figure 2 b). The QDs show two bands in all cases, corresponding to the first and second overtone of the longitudinal optical phonon (LO) of CdS at 305 and 605 cm^−1^, respectively.^24^ Shoulders on either side of the LO peak were observed due to CdO on the CdS surface at high pH, consistent with previous reports.^3^, ^25^ Although Cd^0^ does not show Raman bands, Cd nanoparticles around 5 nm in size (as well as analogous Ag‐based clusters) exhibit collective vibrations that give rise to appreciable bands in the low frequency region at 115 cm^−1^.^26^, ^27^, ^28^, ^29^ Such a peak is observed after 2 min of irradiation using CdS/CdO_*x*_ QDs, which is believed to arise from Cd^0^ localised at the particle surface (Figure 2 c, note that the low resolution of this peak is due to its location at the edge of the spectral window).^23^ At 5 and 10 min of irradiation, the peak is less pronounced, which is assigned to agglomeration after photocatalysis. This agrees with the observation of particle agglomerates in TEM images (Figure 2 d).

Based on these experiments, the route to O_2_‐stabilisation in this system is summarised in Figure 2 e. The consecutive processes are illustrated as (1) light absorption, (2) donor oxidation and (3) proton reduction. As reactions (1) and (2) are substantially faster than (3) in strongly alkaline conditions, excited electrons can accumulate on the particle surface in the form of Cd^0^ sites. Formation of Cd^0^ causes the QDs to agglomerate, which significantly lowers activity. O_2_ provides an easily reduced secondary substrate in the photoreactor that precludes the accumulation of Cd^0^ and thereby maintains the stability of the particle during photocatalysis. Note that this mechanism consumes a portion of electron donor without concomitant release of H_2_.

Taking this mechanism into account, a photocatalytic system was designed where the continued presence of O_2_ was exploited to stabilise the rate of photocatalysis. A constant flow of air was introduced into a photoreactor containing QDs in 5 m KOH with EtOH (7.5 m) as an electron donor and a cobalt co‐catalyst. The vial was irradiated and the outlet gas‐stream was continuously analysed by gas chromatography. The rate of H_2_ evolution reached a maximum activity of 432 mmol${}_{H{}_{2}}$ g^−1^ h^−1^; the highest reported rate for photocatalysis driven by an organic oxidation reaction on CdS under AM 1.5 G, 100 mW cm^−2^ irradiation (to the best of our knowledge higher activity has only been reported when using a S^2−^ donor).^19^ Here, the constant influx of O_2_ was able to stabilise H_2_ production relative to an N_2_‐purged equivalent that operated at only 202 mmol${}_{H{}_{2}}$ g^−1^ h^−1^ (Figure 3 a). MeOH‐promoted H_2_ evolution was similarly high, exhibiting a rate of 165 mmol${}_{H{}_{2}}$ g^−1^ h^−1^ under air (Figure 3 b).

**Figure 3:**
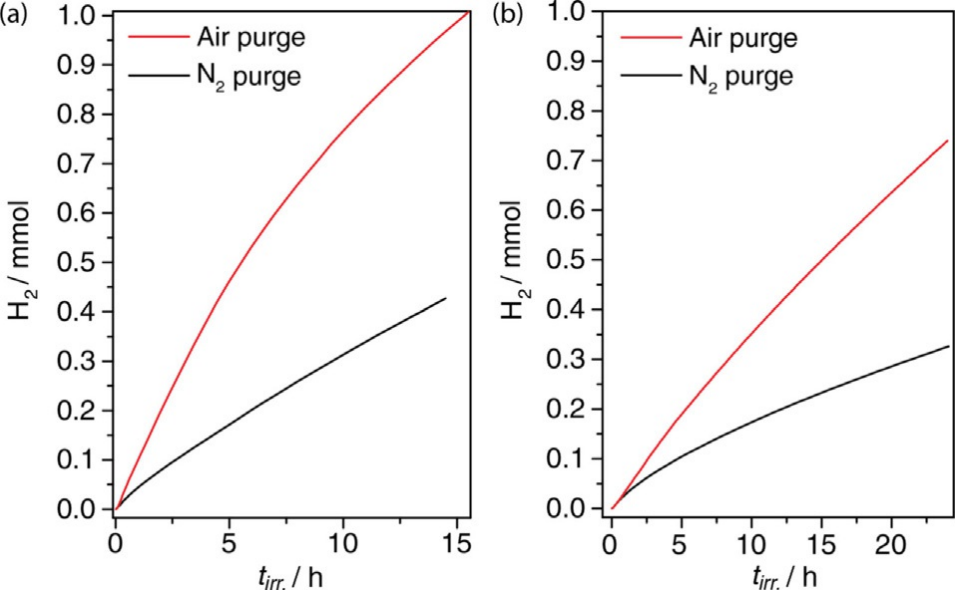
Photocatalytic H_2_ evolution activity from a solution of CdS/CdO_*x*_ (0.5 μm) in 5 m KOH, Co(BF_4_)_2_ (0.25 mm) and (a) EtOH (7.5 m) or (b) MeOH (10 m). In each case the photoreactor was irradiated (AM 1.5 G, 100 mW cm^−2^ at 25 °C) whilst being purged with constant flow of air or N_2_ gas at 3 mL min^−1^.

In summary, the presented system demonstrates how photoredox reactions can be tuned to ensure discharging of potentially inhibiting deactivation reactions. This counter‐intuitive strategy employs O_2_ to regenerate/stabilise a damaged photocatalyst and has achieved unmatched rates of proton reduction driven by photoreforming of an organic substrate. Consideration of aerobic strategies to benefit photocatalysis may therefore be vital in attaining both fast and stable systems in solar fuel and organic photoredox catalysis, where the presence of O_2_ is often seen as a source of inhibition.

## Conflict of interest

The authors declare no conflict of interest.

## Supporting information

As a service to our authors and readers, this journal provides supporting information supplied by the authors. Such materials are peer reviewed and may be re‐organized for online delivery, but are not copy‐edited or typeset. Technical support issues arising from supporting information (other than missing files) should be addressed to the authors.

Supplementary
