# Supplementary material for: Aerobic Conditions Enhance the Photocatalytic Stability of CdS/CdOx Quantum Dots
Source: Chemistry. 2018 Jun 27;24(69):18385–8. doi: 10.1002/chem.201802353 (PMC6348374; doi:10.1002/chem.201802353)
Supplement: Supplementary file 1 — Supplementary [file CHEM-24-18385-s001.pdf]

CHEMISTRY
A European Journal

Supporting Information

Aerobic Conditions Enhance the Photocatalytic Stability of CdS/CdO_x Quantum Dots

David W. Wakerley, Khoa H. Ly, Nikolay Kornienko, Katherine L. Orchard, Moritz F. Kuehnel, and Erwin Reisner*[a]

chem_201802353_sm_miscellaneous_information.pdf

EXPERIMENTAL SECTION

Reagents. All reagents were attained from commercial sources and used without further purification. Semiconductor-grade KOH (99.99%) was used.

Methods and Instrumentation. O_2/N_2 mixtures were generated using a mass flow controller (Brooks Instruments). ^1H NMR spectra were recorded on a Bruker 400 MHz spectrometer at room temperature. UV-Visible spectroscopy was undertaken on a Varian Cary 50 UV-vis spectrometer using a quartz cuvette. Transmission electron microscopy (TEM) images were collected using a FEI Philips Tecnai 20 microscope, with 200 kV accelerating voltage.

Synthesis of oleic acid-capped CdS quantum dots. Oleic acid-capped CdS quantum dots (CdS-OA) were synthesized according to a reported procedure.[1] A mixture of CdO (0.64 g) and oleic acid (OA, 29 mL) in octadecene (89 mL) were heated under an Ar atmosphere to 280°C. Separately, a solution of sulphur (0.08 g) in octadecene (20 mL) was prepared. Half of the sulphur solution was then added rapidly and the other half was added continuously over the course of 2 minutes, after which the vessel was cooled to 220 °C with N_2 gas and then rapidly cooled in a water bath. The solution was diluted with 1:1 hexane:methanol (100 mL) and the particles were precipitated using excess acetone (approximately 300 mL). Isolation of the particles was achieved by centrifugation at 5000 RPM for 3 min, after which they were re-dispersed in hexane. Two further washing steps were carried out by dispersion in hexane and precipitating with acetone, before finally dispersing in hexane.

Synthesis of ligand-free CdS quantum dots. Ligand stripping of CdS-OA was carried out according to a literature procedure to form ligand-free CdS QDs (CdS-BF_4).[2,3] CdS-OA in hexane (5 mL) was reduced to dryness and re-dispersed in a mixture of anhydrous $CHCl_3$ (15 mL) and anhydrous DMF (1.9 mL) under a N_2 atmosphere. The solution was then stirred in the presence of a 9 mL Et_3OBF_4 (1.0 M in acetonitrile) for 1 hour. Me_3OBF_4 (1 M in hexane) was added slowly until the particles precipitated (*c.a.* 3 mL). The precipitate was collected by centrifugation (6000 RPM, 3 min), and re-dispersed in a minimum of DMF and kept < 5°C. The QD concentration was determined from UV-visible spectroscopy based on the position and absorbance of the absorption maximum around 450 nm.[4] Full characterisation details (XRD, UV-visible absorption) can be found elsewhere.[3,5]

Photocatalytic H_2 evolution. Photocatalysis was carried out in a pyrex photoreactor thermostated at 25 °C. Solar irradiation was emulated by a solar light simulator (Newport Oriel, adjusted to irradiate 100 mW cm^{-2} photon intensity onto the photoreactor) equipped with an air mass 1.5 global (AM 1.5G) filter and a water filter (10 cm path length) to remove IR radiation.

In a typical experiment CdS-BF_4 QDs in DMF (1 nmol) were transferred to a photoreactor and the DMF was removed at room temperature *in vacuo* while stirring. The electron donor and 2 mL of various concentrations of KOH were then added followed by 10 µL of an aqueous $Co(BF_4)_2$ solution (50 mM). The photoreactor was sealed with a rubber septum and purged with the desired gas mixture for 10 min, after which the vial was irradiated whilst stirring at 600 RPM. If aerobic conditions were required, the samples were left unpurged. To guarantee safe operation in the presence of O_2, we employed a small photoreactor and kept the photoreactor away from sources of ignition at all times. The accumulation of H_2 was quantified through periodic headspace gas analysis (50 µL) by gas chromatography. Experiments were repeated in triplicate and are given as a mean ± standard deviation, calculated as described below.

Photocatalysis under a constant flow of N_2 or air was undertaken in the photocatalytic set-up described above with a gas inlet into the reaction solution of the photoreactor. Gas was purged into the vial at a constant flow of 3 mL^{-1} min^{-1} using a mass flow controller (Brooks Instruments). An outlet from the headspace provided a constant measure of the H_2 evolution via gas chromatography.

Treatment of data. All analytical measurements were performed in triplicate and are given as the unweighted mean ± standard deviation (σ). σ of a measured value was calculated using equation (1).

$$\sigma = \sqrt{\frac{\sum(x-\bar{x})^2}{n-1}} \qquad \text{(Eq. 1)}$$

Where n is the number of repeated measurements, x is the value of a single measurement and $\bar{x}$ is the unweighted mean of the measurements.

The activity per weight of catalyst ($mol_{H_2}\ g_{cat.}^{-1}\ h^{-1}$) was calculated using equation 2 from the molar weight of the QD and co-catalyst.

$$\text{Activity } (mol_{H_2}\ g_{cat.}^{-1}\ h^{-1}) = \frac{n_{H_2}}{4/3\pi r_{CdS}^3 N_a \rho_{CdS} n_{QD} + n_{co-cat} M_{co-cat}} \div t_{irr.} \qquad \text{(Eq. 2)}$$

Where n_{H_2} is the H_2 produced (mol), r_{CdS} is the radius of the QD (cm), ρ_{CdS} is the density of CdS (4.84 g cm^{-3}), N_a is Avogadro's number (mol^{-1}), n_{QD} is the number of moles of QD (mol), $n_{co\text{-}cat.}$ is the number of moles of added co-catalyst, $M_{co\text{-}cat.}$ is the mass of co-catalyst (taken as 232.54 g mol^{-1} for $Co(BF_4)_2$), $t_{irr.}$ is the irradiation time of the sample (h).

Gas analysis. Gas chromatography (GC) was carried out on an Agilent 7890A gas chromatograph with a thermal conductivity detector. H_2 was analysed using a HP-5 molecular sieve column (0.32 mm diameter) and N_2 carrier gas with a flow rate of approximately 3 mL min^{-1}. The GC oven temperature was kept at 45 °C in both cases. Methane (2% CH_4 in N_2) was used as internal standard or external standard after calibration with different mixtures of known amounts of $H_2/O_2/CH_4$.

Transient absorption spectroscopy. TA spectra were obtained using an Ultrafast Systems Helios TA system with a Coherent Libra amplified Ti:sapphire laser system and Coherent OPerA optical parametric amplifier (OPA) pump/probe source. The samples were excited with ~50-fs laser pulses generated by the OPA at a repetition rate of 1.1 kHz. TA spectra were obtained by time-delaying a broadband supercontinuum probe pulse that is overlapped in time and space with the femtosecond pump pulse. The supercontinuum is produced by focusing a small portion of the amplified laser fundamental into a sapphire plate. Multiwavelength TA spectra were recorded using dual spectrometers (signal and reference) equipped with fast Si array detectors. In all experiments, the excitation was achieved with 430 nm light at a power of 1 µJ. The data was fit with a multi-exponential decay function:

$I(t) = A_1 * e\text{^}(-k_1 * t) + A_2 * e\text{^}(-k_2 * t) \ldots + \text{baseline}.$

Raman analysis. Raman spectra were recorded using a confocal Raman spectrometer LabRam (Horiba Jobin Yvon) equipped with a liquid-nitrogen-cooled Symphony CCD detector (Horiba Jobin Yvon). A 514.73 nm line of an Ar ion laser (Coherent Innova 300c) was used as excitation wavelength. 10 µM solutions of CdS-BF_4 QDs in 10 M KOH were measured in a rotating quartz cuvette with and without added EtOH. Accumulation time of the Raman spectra was 60 seconds. The laser power was set to 5 mW. The laser light was focused into the solution using a 20x objective (Nikon, 20x NA 0.5). For excitation, the 413 nm laser line of the Kr ion laser (Sabre) was used.

Supporting Tables

Table S1. Anaerobic and aerobic experiments with CdS/CdO_x in various concentrations of KOH[a]

[KOH] / M	Time / h	H_2 under N_2/ µmol	± σ / µmol	H_2 under air / µmol	± σ / µmol
0.05	0.00	0.00	N/A	0.00	N/A
	0.50	0.14	0.01	0.01	0.01
	1.00	0.46	0.02	0.03	0.03
	2.00	1.32	0.09	0.03	0.02
	3.00	2.38	0.19	0.04	0.02
	4.00	3.60	0.16	0.07	0.06
	5.00	4.75	0.15	0.11	0.09
	6.00	5.58	0.72	0.15	0.13
	7.00	6.68	0.83	0.22	0.16
	20.00	21.76	1.49	3.83	1.05
0.5	0.00	0	N/A	0	N/A
	0.50	0.64	0.05	0.083	0.01
	1.00	2.39	0.43	0.190	0.05
	2.00	7.77	1.51	0.59	0.18
	3.00	13.48	1.89	1.74	0.39
	4.00	19.00	2.40	3.09	0.77
	5.00	24.09	3.02	4.86	1.02
	6.00	29.27	2.56	6.92	1.64
	7.00	28.93	5.30	9.42	2.01
	20.00	79.73	8.36	38.39	14.60
5	0.00	0.00	N/A	0.00	N/A
	0.50	8.91	1.29	7.28	1.66
	1.00	18.64	2.45	19.25	2.36
	2.00	35.35	5.79	43.10	0.35
	3.00	47.84	7.13	65.29	0.45
	4.00	58.30	8.89	82.30	1.46
	5.00	68.14	9.53	91.96	10.88
	6.00	78.01	12.29	105.61	10.00
	7.00	81.82	7.42	115.99	6.87
	20.00	136.54	10.74	167.05	7.56

[a]CdS/CdO_x quantum dots (4.26 nm, 0.5 µM) irradiated with simulated solar light (AM 1.5G, 100 mW cm^{-2}), with 2 mL KOH (5 M), 10 M MeOH and 0.25 mM $Co(BF_4)_2$ at room temperature in a sealed photoreactor with an internal volume of 7.91 mL.

Table S2. Control experiments[a]

Control description	[KOH] / M	[MeOH] / M	Time / h	H_2 / μmol	σ / %
No QDs dots	10.0	1.0	1.0	0.0	N/A
No light[b]	10.0	1.0	1.0	0.0	N/A
No MeOH	10.0	0.0	24.0	2.54	0.05

[a]Samples irradiated with simulated solar light (AM 1.5G, 100 mW cm^{-2}), with CdS/CdO_x quantum dots (0.5 μM, except when no dots were added) with 2 mL KOH (10 M) and 0.25 mM $Co(BF_4)_2$ at room temperature in a sealed photoreactor under anaerobic conditions with an internal volume of 7.91 mL. [b]Samples were wrapped in foil and stirred in the dark.

Table S3. Dependence of O_2 level on photocatalytic H_2 evolution[a]

**% O_2 in headspace**	**Time / h**	**H_2 / µmol**	**σ / µmol**	**O_2 / %**	**σ / %**
0	0	0.00	N/A	-	-
	1	12.87	0.96	-	-
	2	25.02	2.00	-	-
	3	34.58	2.72	-	
	4	42.927	2.92	-	-
	24	106.97	2.52	-	-
10	0	0.00	N/A	-	-
	1	14.70	1.05	-	-
	2	31.47	2.36	-	-
	3	46.50	3.58	-	-
	4	61.53	2.98	-	-
	24	147.00	2.21	-	-
21	0	0.00	N/A	20.97[b]	-
	1	13.38	0.59	10.27	0.31
	2	27.89	1.75	2.21	1.05
	3	40.80	2.54	0.00	0.00
	4	53.76	2.77	0.00	0.00
	24	153.87	2.39	0.00	0.00
50	0	0	N/A	-	-
	1	11.01	4.04	-	-
	2	23.15	5.08	-	-
	3	35.10	5.14	-	-
	4	46.87	5.46	-	-
	24	168.93	4.34	-	-
100	0	0	N/A	-	-
	1	7.35	0.73	-	-
	2	15.40	2.25	-	-
	3	23.42	3.37	-	-
	4	32.16	5.39	-	-
	24	184.05	7.72	-	-

[a]CdS/CdO_x quantum dots (5.19 nm, 0.5 µM) irradiated with simulated solar light (AM 1.5G, 100 mW cm^{-2}), with 2 mL KOH (5 M), 12.35 M MeOH and 0.25 mM $Co(BF_4)_2$ at room temperature in a sealed photoreactor with an internal volume of 7.91 mL. [b]O_2 level assumed to be atmospheric before initiating photocatalysis.

Table S4. Electron donor dependence on aerobic *vs.* anaerobic H_2 evolution[a]

Electron donor	Time / h	H_2 under N_2/ µmol	σ / µmol	H_2 under air/ µmol	σ / µmol
MeOH	0	0.00	N/A	0.00	N/A
	1	10.27	1.65	9.69	1.29
	2	18.25	2.61	19.72	2.08
	3	25.87	3.27	28.56	2.53
	4	31.62	3.66	36.91	2.91
	24	95.91	4.31	124.96	6.46
EtOH	0	0.00	N/A	0.00	N/A
	1	30.00	3.42	29.45	5.58
	2	45.92	3.28	49.94	9.21
	3	57.41	1.54	66.59	11.29
	4	71.28	10.97	82.70	12.45
	24	153.35	12.44	166.49	7.71
TEOA	0	0	N/A	0	N/A
	1	0.73	0.26	0.35	0.05
	2	0.96	0.38	0.49	0.08
	3	1.13	0.48	0.57	0.1
	4	1.24	0.53	0.63	0.18
	24	4.65	0.93	2.28	0.26

[a]CdS/CdO_x quantum dots (5.19 nm, 0.5 µM) irradiated with simulated solar light (AM 1.5G, 100 mW cm^{-2}), with 2 mL KOH (5 M), 1 M electron donor and 0.25 mM $Co(BF_4)_2$ at room temperature in aerobic conditions in a sealed photoreactor with an internal volume of 7.91 mL.

Supporting Figures

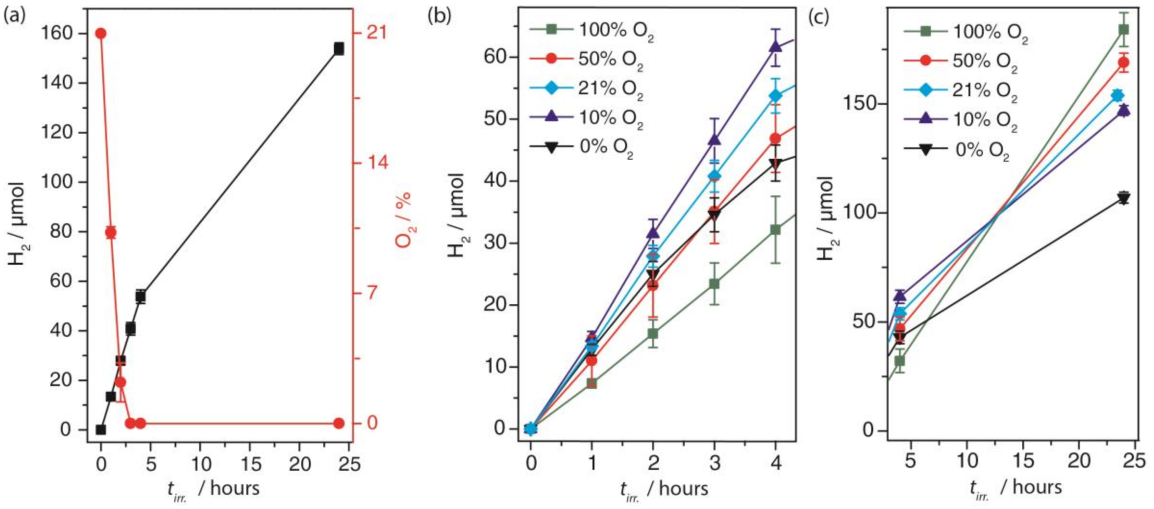

Figure S1. (a) Photocatalytic H_2 evolution (AM1.5 G, 100 mW cm^{-2}) of CdS/CdO_x QDs (0.5 µM) in 5 M KOH (2 mL) with 0.25 mM $Co(BF_4)_2$ and MeOH (12.35 M) in air with concurrent decrease in O_2 level. The volume of the closed photoreactor was 7.91 mL. The effect of the initial O_2 % in the aforementioned conditions under various atmospheres of O_2 between (b) 0-4 hours and (c) 4-24 hours.

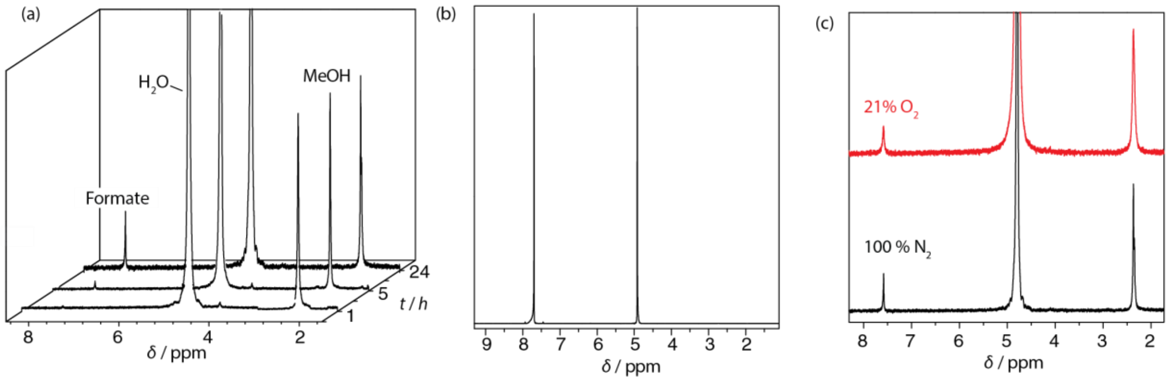

Figure S2. ^{1}H-NMR spectra of 10 M NaOD/D_2O containing 0.1 M MeOH with CdS/CdO_x QDs (0.5 µM) and $Co(BF_4)_2$ (0.25 mM) (a) after specified periods of irradiation (AM1.5G, 100 mW cm^{-2}) at 25 °C under anaerobic conditions. (b) Reference spectrum of NaCOOH in 10 M NaOH/D_2O. (c) ^{1}H-NMR spectrum under aerobic conditions or anaerobic conditions after 24 hours.

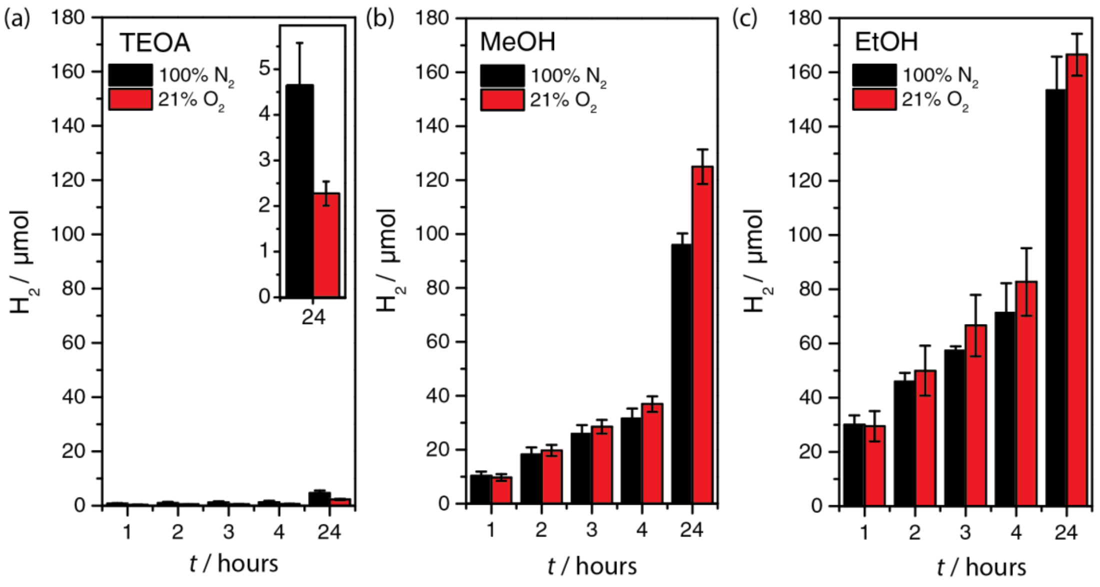

Figure S3. The photocatalytic H_2 evolution activity (AM1.5 G, 100 mW cm^{-2}) of CdS/CdO$_x$ QDs (0.5 μM) in 5 M KOH (2 mL) with 0.25 mM $Co(BF_4)_2$ and (a) 1 M TEOA, (b) 1 M MeOH, (c) 1 M EtOH under air/N_2. The volume of the closed photoreactor was 7.9 mL.

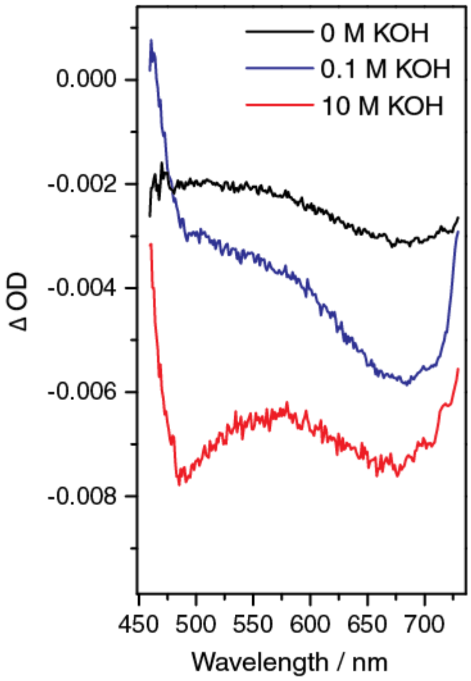

Figure S4. TA spectra at ~4.5 ps of CdS QDs in aerobic conditions with ethanol as a sacrificial donor. Lower magnitude and proportionally weaker band edge bleach under lower pH solutions points to increasing rates of charge trapping and recombination under these conditions.

Supporting References

[1] L. Huang, X. Wang, J. Yang, G. Liu, J. Han, C. Li, *J. Phys. Chem. C* **2013**, *117*, 11584–11591.

[2] E. L. Rosen, R. Buonsanti, A. Llordes, A. M. Sawvel, D. J. Milliron, B. A. Helms, *Angew. Chem. Int. Ed.* **2012**, *51*, 684–689.

[3] C. M. Chang, K. L. Orchard, B. C. M. Martindale, E. Reisner, *J. Mater. Chem. A* **2016**, *4*, 2856–2862.

[4] W. W. Yu, L. Qu, W. Guo, X. Peng, *Chem. Mater.* **2003**, *15*, 2854–2860.

[5] D. W. Wakerley, M. F. Kuehnel, K. L. Orchard, K. H. Ly, T. E. Rosser, E. Reisner, *Nat. Energy* **2017**, *2*, 17024.

End of Supporting Information
